# Reciprocal regulation of microRNA and mRNA profiles in neuronal development and synapse formation

**DOI:** 10.1186/1471-2164-10-419

**Published:** 2009-09-08

**Authors:** Sergei A Manakov, Seth GN Grant, Anton J Enright

**Affiliations:** 1 Genes to Cognition Programme, The Wellcome Trust Sanger Institute, Hinxton, Cambridge, CB10 1SA, UK; 2 European Bioinformatics Institute, Hinxton, Cambridge, CB10 1SD, UK

## Abstract

**Background:**

Synapse formation and the development of neural networks are known to be controlled by a coordinated program of mRNA synthesis. microRNAs are now recognized to be important regulators of mRNA translation and stability in a wide variety of organisms. While specific microRNAs are known to be involved in neural development, the extent to which global microRNA and mRNA profiles are coordinately regulated in neural development is unknown.

**Results:**

We examined mouse primary neuronal cultures, analyzing microRNA and mRNA expression. Three main developmental patterns of microRNA expression were observed: steady-state levels, up-regulated and down-regulated. Co-expressed microRNAs were found to have related target recognition sites and to be encoded in distinct genomic locations. A number of 43 differentially expressed miRNAs were located in five genomic clusters. Their predicted mRNA targets show reciprocal levels of expression. We identified a set of reciprocally expressed microRNAs that target mRNAs encoding postsynaptic density proteins and high-level steady-state microRNAs that target non-neuronal low-level expressed mRNAs.

**Conclusion:**

We characterized hundreds of miRNAs in neuronal culture development and identified three major modes of miRNA expression. We predict these miRNAs to regulate reciprocally expressed protein coding genes, including many genes involved in synaptogenesis. The identification of miRNAs that target mRNAs during synaptogenesis indicates a new level of regulation of the synapse.

## Background

MicroRNAs (miRNAs) are known to regulate the expression of target genes both at the level of mRNA translation and mRNA stability [1,2]. This ability to influence multiple genes makes miRNAs well suited for the regulation of systems where the expression of large numbers of genes changes in concert. Coordinated waves of mRNA expression are well described during embryonic development [3] including in brain development and synaptogenesis *in vitro *[4,5] and thus many genes may therefore be targets for miRNAs. Consistent with this role, miRNAs are differentially regulated in brain [6-9] and miRNA deficient vertebrates have severe abnormalities in brain development [3]. In mouse brain development, the period of neurogenesis between embryonic days 9-17 is followed by postmitotic neurons extending neurites and ultimately forming synapses and networks [10]. Whole genome mRNA profiling of primary cultures from E17.5 embryos over three weeks shows simultaneous regulation of a set of genes encoding synaptic proteins [4]. The extent to which these synaptic genes are regulated by miRNAs is unknown.

Previous studies have demonstrated that miRNA regulation is measurable at both the mRNA and protein level of targeted genes [2,11]. At the same time, computational miRNA target analysis has improved by combining mRNA and miRNA expression profiling [12]. We therefore sought to concomitantly profile miRNA and mRNA during synaptogenesis and use computational methods to examine the potential regulatory functions of miRNAs.

## Results

### mRNA Expression Analysis

Previous studies of neuronal network activity in mouse E17.5 primary neuronal cultures established that spontaneous firing arises after three to six days and that during this phase there is a wave of synthesis of mRNAs encoding synaptic proteins [4]. We therefore plated primary neuronal cultures from mouse forebrain of E17.5 day embryos, extracted total RNA (days *in vitro *(DIV) 1, 2, 4, 8) and profiled mRNA levels on DNA microarrays. Array data was deposited in ArrayExpress [13] under accession E-TABM-615. A total of 23,195 microarray probes passed the differential expression significance threshold (see Methods). Their expression profiles were clustered using the Markov Cluster algorithm (MCL) [14,15], identifying 11 clusters. The two largest captured over 75% of the probes passing the differential expression threshold.

**Figure 1:**
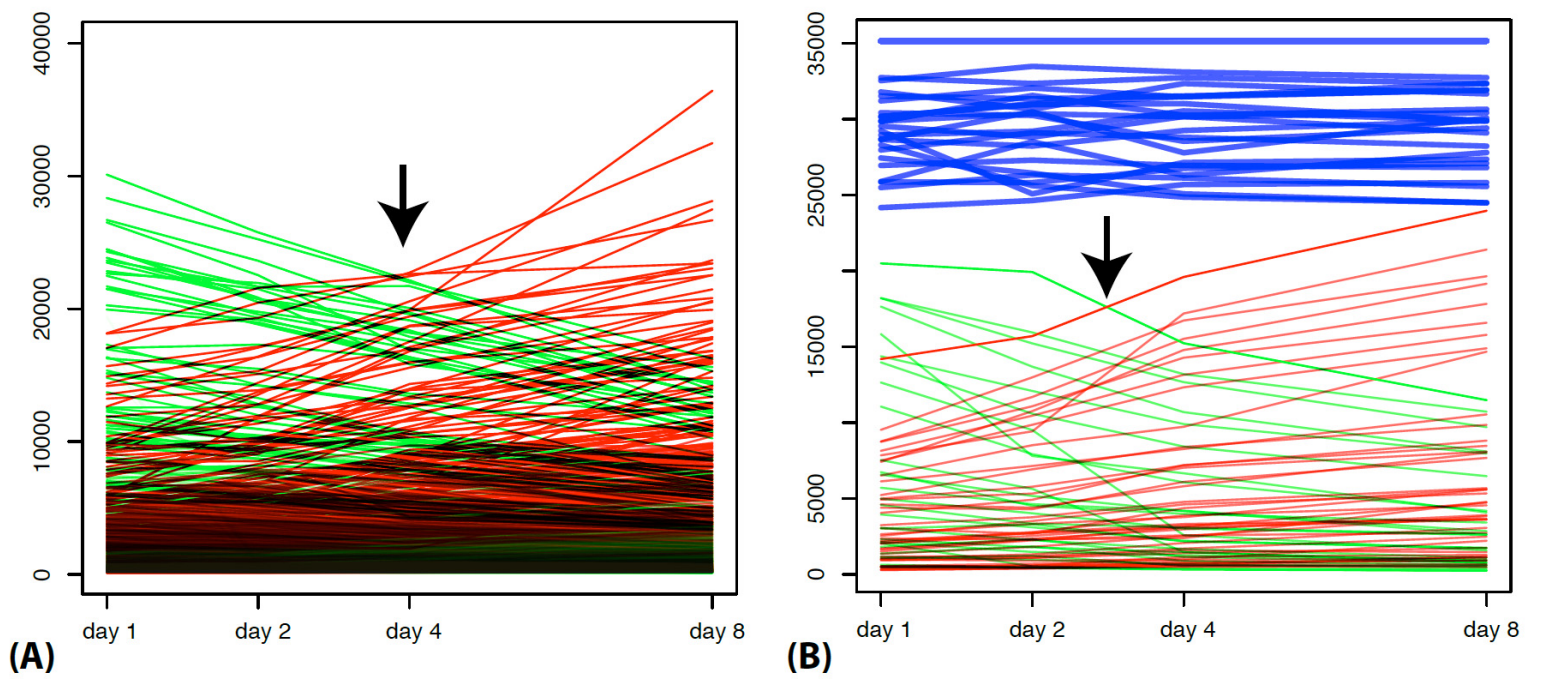
**mRNA and miRNA profiles in neuronal primary culture development**. Intensities of mRNA and miRNA microarray probes across eight days of *in vitro *development. (*A*) Intensities of 2993 out of 3000 of the most differentially regulated mRNA microarray probes. Probes from expression clusters displaying a decrease in signal across all timepoints are in green and those displaying an increase are in red. (*B*) Intensity of 101 miRNA microarray probes showing significant down-regulation (green), and of 97 probes showing up-regulation (red). Intensities of 25 out of 30 probes with the highest intensities is in blue.

Probes in these two clusters had, on average, opposite expression profiles, i.e. signals from the probes in one cluster were decreasing through the course of eight days, while the signal from the probes in the second cluster were increasing (Figure 1A). Probes in the two clusters were mapped to 4,620 and 4,405 genes respectively (see Methods). In these two groups of genes we assessed enrichment of Gene Ontology (GO) [16] terms (see Methods) and enrichment of genes encoding the post synaptic proteome (PSP) [17-19].

Signs of a defined developmental program emerged, which agreed with results from a previous hippocampal cultures study [4]. We found that GO terms enriched in the cluster of down-regulated genes were related to nuclear localisation, DNA biosynthesis, chromatin assembly, cell cycle, RNA and protein biosynthesis, and regulation of various metabolic processes. On the other hand, GO terms overrepresented in up-regulated genes were mostly involved in ion transport, secretion, synaptic transmission and ATP biosynthesis, while enriched cellular component GO terms were cytoplasmic, synaptic and membrane (for the list of enriched GO terms see Additional file 1). Up-regulated genes were also highly significantly enriched in postsynaptic proteome (PSP) genes (*P *< 5.86*e *- 19).

### miRNA Expression Analysis

In order to reliably relate mRNA and miRNA expression, the same total RNA samples were used for miRNA profiling. Array data was deposited in the ArrayExpress [13] under accession number E-TABM-618. In this way 238 miRNAs out of 380 miRNAs profiled by microarray were defined as significantly differentially regulated with no magnitude of change cutoff (a loose definition of differential expression) while 105 miRNAs were up or down regulated more than 1.5 fold (a stringent definition, see Methods for details and Additional file 2 and 3 for the complete list of differentially expressed miRNAs).

All significantly differentially expressed miRNAs were separated by MCL [14,15] into nine expression clusters. The expression profiles of differentially expressed miRNAs are similar to the profiles of mRNA coding genes (see above). Two large clusters encompassed the majority (83.2%) of clustered miRNAs. Under the loose definition of differential expression each of these two clusters contained approximately 100 miRNAs, which was 7.4 times more than any of the other clusters. As with mRNA expression, the average profile of the two largest miRNA expression clusters displayed opposite trends. We noted that up-regulated probes of both mRNA and miRNA mi-croarray probes intersect around day four (shown with an arrow, Figure 1). Coincidently, this is the time electrical activity can first be detected in primary cultures [4,20].

Surprisingly, miRNAs expressed at high levels were found to be only rarely differentially expressed (Figure 1B). Even under the loose definition of differential expression, among the 30 most highly expressed miRNAs, only six show any significant differential regulation, while 24 are expressed at steady-state levels (hypergeometric *P *≤ 1.28*e *- 07 for depletion). We considered these highly steady-state expressed miRNAs to be a distinct category of miRNAs.

In summary, for the purpose of the analysis of miRNAs described in this study we assumed that there are three major categories of miRNAs expressed during the development of neuronal cultures: Up- and down-regulated, and those expressed at high levels in a steady state fashion. For the annotated list of the 30 most highly expressed miRNAs, and lists of the up- and down-regulated miRNAs see Additional file 2 and 3.

When dealing with microarray data there is always a concern that technical biases, such as variability in hybridization efficiencies and other chip artifacts, may influence the results. Therefore we randomly picked a highly steady-state expressed miRNA and a relatively lowly expressed member of each of the two major categories of differentially expressed miRNAs and profiled their expression by Taqman qRT-PCR. The qRT-PCR data agree well with microarray data for the three cases that were tested (see Methods and Additional file 4).

### Role of highly expressed miRNAs

The expression analysis described above provided insights into miRNA expression but not necessarily function. In order to detect functional effects of these miRNA signatures we utilised miRNA seed analysis. Positions 2 to 8 of the 5'end of a mature miRNA have been shown to be most critical for defining specificity [21], and we refer to this particular region as a miRNA seed in this study.

The distribution of miRNA seed matching sites (7-mer "words") was assessed in 3'UTRs using Sylamer [22](see Methods). Words complementary to seed regions of all profiled miRNAs were analysed across the 3'UTRs of all of the genes profiled in our experiments. One current view of the function of miRNAs is that they not only modulate protein levels but frequently also trigger the degradation of mRNAs upon binding to 3'UTRs of target mRNA transcripts [1]. Thus observing significant extremes in the distribution of words in 3'UTRs of transcripts sorted on average expression levels may indicate biological activity of corresponding miRNAs [23,24].

**Figure 2:**
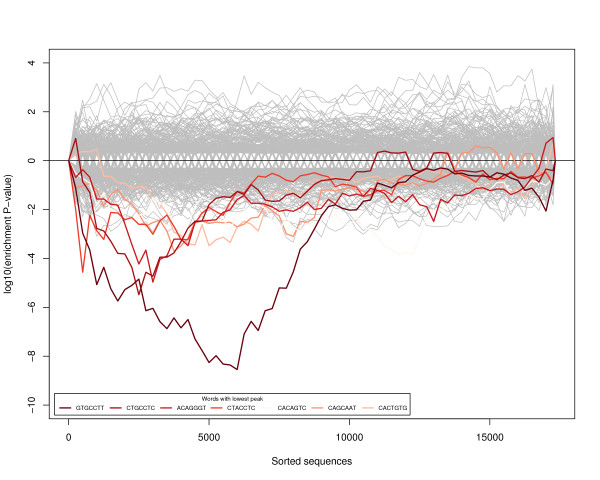
**Distribution of sites complementary to profiled miRNA seed regions**. We analyzed distributions of words complementary to the seed regions of profiled miRNAs (positions 2 - 8 of mature sequences) in 3'UTRs of 17,333 profiled genes. Genes are sorted according to the average expression (from highest expressed to lowest expressed). Probability values are plotted with a positive sign for enrichment and with negative sign for depletion. Six out of seven most significantly depleted words in 3'UTRs profiled are shown in shades of red. The most significant depleted word "GTGCCTT" is complementary to positions 2 - 8 of mmu-miR-124. The second most depleted word has a central 5-mer "TGCCT" which is complementary to positions 3 - 7 of mmu-miR-124. The central 5-mer of the third most depleted word is complementary to positions 2 - 6 of both mmu-miR-125a-5p and mmu-miR-125b-5p. The fourth most depleted word "CTACCTC" is complementary to positions of 2 - 8 of eight miRNAs of mmu-let-7 family. The sixth most depleted word contains a 6-mer complementary to positions 3 - 8 of mmu-miR-137, and the seventh to positions 2 - 8 of miR-128.

We found that several miRNA specific words displayed significant depletion in 3'UTRs of highly expressed genes (Figure 2). The most depleted (*P *< 2.85*e *- 09) and the second most depleted (*P *< 3.28*e *- 06) words in 3'UTRs of highly expressed genes were entirely or partially complementary to the seed region of mmu-miR-124 ("AAGGCAC"). This miRNA was previously implicated in the regulation of neuronal differentiation [25-29] and has been suggested to have a function in maintaining neuronal identity [1]. We found that other words among the six most depleted corresponded to the seed regions of 12 more steady-steate highly expressed miRNAs: The mmu-miR-125 family, mmu-miR-137, mmu-miR-128 and the mmu-let-7 family. These miRNAs were previously reported to be highly expressed in mammalian brain and to be involved in differentiation of neuronal progenitors [6,29-33]. Our result adds to this notion by suggesting that the global function of these miRNAs in neuronal cultures is closely related and complementary to that of mmu-miR-124.

**Figure 3:**
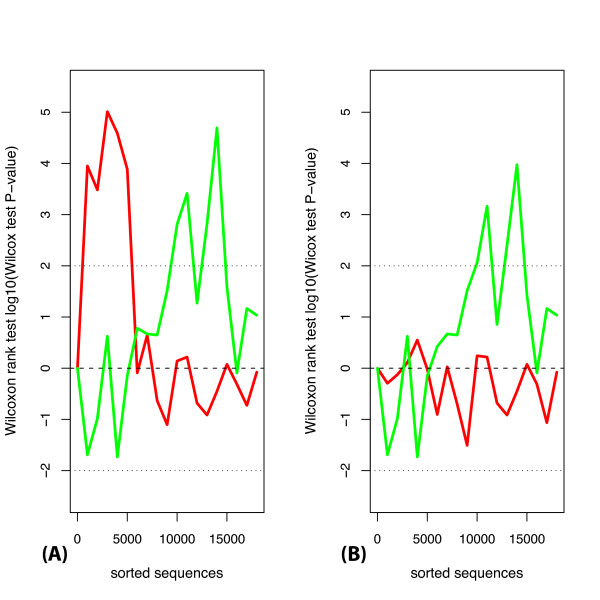
**Expression levels of miRNAs corresponding to the 10 most depleted or 10 most enriched seed matching sites**. We assessed the expression levels of miRNAs with seed-regions complementary to the 10 most depleted (red line) or 10 most enriched words (green line) in 3'UTRs of 17333 profiled genes. Genes are sorted according to average expression (from highest to lowest). The p. values are plotted with a positive sign if the subset of miRNAs is expressed higher than the average level of miRNA expression, and with a negative sign if lower. The subset of miRNAs with seeds complementary to the 10 most depleted words in the beginning of the sorted list (i.e. in 3'UTRs of highly expressed genes) were on average highly expressed (A, red line). If seed matching sites of 12 miRNAs steady-state highly expressed miRNAs (Figure 2) were subtracted, the derived subset of miRNAs was expressed at the average level (B, red line). miRNAs corresponding to the 10 most enriched words (green line) in the second half of the list (i.e. in 3'UTRs of relatively lowly expressed genes) were highly expressed (A, green line). This did not depend on the subtraction of seed matching sites of 12 highly expressed miRNAs (B, green line). Black dotted lines are showing p. value cutoff *-log*_10_(0.01) for higher than average expression and *log*_10_(0.01) for lower than average expression.

To extend the miRNA targeting analysis, instead of using a particular significance threshold we analyzed rankings of miRNAs based on word enrichment/depletion probability values (see Methods). We estimated whether miRNAs corresponding to the 10 most depleted/enriched words were expressed at significantly different levels to all miRNAs on average (Figure 3). Using this method we again found that the words corresponding to 13 miRNAs identified with the word counting method (see above) are depleted in 3'UTRs of highly expressed genes. Interestingly, this depletion was not observable after approximately 6,000 3'UTRs were examined (i.e. moving toward lowly expressed genes). This indicates that targeting by highly expressed at steady-state levels miRNAs is avoided by genes highly expressed during the course of neuronal culture development in this study. On the other hand, genes expressed at lower levels (i.e. below approximately top 6000 genes) may be targeted by this class of miRNAs.

In accordance with this hypothesis, we found seed matching sites for highly expressed miRNAs to be ranked among the most enriched in 3'UTRs of genes expressed at lower levels. For example, when 3'UTRs of approximately 3300 of the most lowly expressed genes were considered, miRNAs corresponding to the 10 most enriched words, were significantly more highly expressed than miRNAs on average (Wilcoxon test *P *< 2*e *- 05, see Methods). Four of the six most highly expressed of these miRNAs were not differentially regulated and were among the top 25 most expressed miRNAs in the neuronal cultures (mmu-miR-137, mmu-miR-9*, mmu-miR-17, mmu-miR-30c). Additionally, we found many other miRNAs with seed matching sites ranked as the most enriched to be expressed significantly higher than the average miRNA expression level, albeit lower than "top 25" level (see Additional file 5 for the list).

Overall we observed the seed matching site depletion of 13 highly expressed miRNAs in 3'UTRs of highly expressed genes and seed matching site enrichment of a wider range of relatively highly expressed miRNAs in the 3'UTRs of lowly expressed genes. These findings indicate that many of the putative targets of highly expressed miRNAs are not among highly expressed typical neuronal genes (i.e. many brain specific and post-synatpic proteome coding genes), but rather among the genes which are expressed at lower levels, if reliably expressed at all. Therefore, many of the putative targets are likely to be non-neuronal genes. For example, genes annotated to "immune response" Gene Ontology (GO) term [16] (304 genes) were very significantly enriched among the 10,000 least expressed genes in neuronal cultures (*P *< 8.80*e *- 27).

To complement the above enrichment analysis, we used traditional target prediction (TargetScan [21]) to look for individual evolutionary conserved putative targets among neuronal genes. By the stage of E17.5 the majority of neuronal progenitors had finished division and differentiated [10]. One might anticipate that miRNAs that are highly expressed at this stage may participate in down-regulation of genes specific to progenitor maintenance. Several signalling pathways were shown to be critically important for maintenance of progenitor division, e.g. the Notch pathway [34], FGF2 pathway [35], Ephrin B1 pathway [36] and several others [37] (see Methods). Some of the genes implicated in these pathways (for example Efnb1) have been shown to be highly expressed in brain during neurogenesis stage, but to be down-regulated afterwards [38], i.e. within the time frame profiled in our experiments. Indeed, conserved target sites of several steady-state highly expressed (in top 30) miRNAs can be found in 3'UTRs of the selected key factors important for maintenance of the progenitor cells in undifferentiated state. Six key factors for progenitor maintenance, including Fgf2, Notch1 and Efnb1, contained such sites (Table 1). Observing evolutionarily conserved seed matching sites for a relatively small category of miRNAs is an indication that these miRNAs may be involved in the regulation of progenitor maintenance genes.

**Table 1:** miRNA targeting of neural progenitor maintenance genes

	**Target Gene**					
**miRNA ID**	**Fgf2**	**Fgfr1**	**Efnb1**	**Rgs3**	**Notch1**	**Mef2d**

**mmu-let-7 **family						(2..8) CUACCUC
**mmu-miR-15b **and **16**	(1..7) GCUGCUA × 2	(2..8) UGCUGCU	(1..7) GCUGCUA			
**mmu-miR-17 **and **20a**			(2..8) GCACUUU			
**mmu-miR-25**				(2..8) GUGCAAU	(2..7) UGCAAU	(2..8) GUGCAAU
**mmu-miR-30c**					(1..7) GUUUACA	
**mmu-miR-103**	(1..8) AUGCUGCU		(2..7) UGCUGC			(2..8) AUGCUGC
**mmu-miR-124**			(2..8) GUGCCUU			
**mmu-miR-125 **family						(1..7) UCAGGGA

Evolutionarily conserved sites complementary to seed regions of highly expressed miRNAs (among top 30 most expressed) were found in 3'UTRs of the genes previously shown to be involved in maintenance of neural progenitors: Fgf2 - fibroblast growth factor 2 [35], Fgfr1 - fibroblast growth factor receptor 1 [35], Efnb1 - ephrin B1 [36], Rgs3 - PDZ-RGS3 [36], Notch1 [34] and Mef2d - myocyte enhancer factor 2D [70]. RNA sequences in uppercase represent the seed matching sites (from TargetScan [21]) of aligned 3'UTRs. All sequences are conserved between human, mouse, rat and dog. Numbers in parentheses show the the positions of mature miRNA sequences to which the 3'UTR sites are complementary.

### Genomic distribution of miRNA genes

It has previously been demonstrated that miRNAs in genomic clusters can be co-expressed [39] and can regulate genes involved in one biological pathway [40]. We sought to investigate if such a phenomenon takes place in neuronal cultures.

105 differentially regulated miRNAs (under strict definition, see Methods) were mapped to 53 miRNA genes with up-regulated expression and 39 genes with down-regulated expression during the developmental timecourse (see Methods). These genes are situated in 14 genomic clusters of size two or larger (with at least one differentially expressed miRNA gene).

We found that only five clusters comprised almost half (47%) of all differentially expressed miRNA genes. Chromosome 12 had two clusters containing 37 and 10 miRNA genes each, approximately 114 Kb apart. These two clusters together included 28 miRNA genes which were up-regulated during the neuronal development. Similarly, the 15 down-regulated miRNA genes were spread between three clusters: A cluster of six genes on the chromosome 14 and two clusters (six and seven genes each) on chromosome X around 300 Kb apart. All genomic clusters were dedicated to one of the expression modes, i.e. under strict definition of differential expression no miRNA genes were inversly expressed within one genomic cluster (see Additional file 6 for the list of miRNA genes in large genomic clusters and their associated genomic features).

A different genomic distribution was found for miRNA genes expressed at steady-state and high levels. The 30 miRNAs most highly expressed at steady-state level (by strict definition) were mapped to 43 miRNA genes. With the exception of one gene (mmu-mir-17 which gives rise to both a steady-state highly expressed mmu-miR-17 and differentially regulated mmu-miR-17*), none were situated in a genomic cluster of more than three miRNA genes. In fact, only seven steady-state highly expressed miRNA genes (approximately 16%) genes were in clusters containing more than two genes. Around 29% of all miRNA genes were situated in clusters of that size or bigger. Thus, miRNA genes which are highly expressed at steady-state levels in neuronal cultures are under-represented in genomic clusters of more than two (hypergeometric depletion *P *< 0.03) or three (*P *< 2.3*e *- 4) miRNA genes.

We examined genomic features and potential regulatory sites of the miRNA clusters described above using the miRBase genomics website [41]. Of the five largest genomic clusters (see Additional file 6) three showed significant evidence for co-transcription and one also had an experimentally determined promoter associated regulatory motif.

### Relation of the seeds of differentially expressed miRNAs

Co-regulation of differential expression and co-localization of a large number of miRNAs in the genome suggests to us that such miRNAs are likely to be functionally related [21]. In order to further test this we looked at the relationship between miRNA seed sequences in the two categories of differentially expressed miRNAs. We obtained a list of conserved miRNA families based on miRNAs sharing common seed regions (positions 2 - 8). We then defined families as being differentially expressed if any member of that family was differentially expressed in our study. We noted, that while many miRNA families had more than one member, we have not found a single family containing both up-and down-regulated miRNAs under the strict definition of differential expression.

To highlight the relation between the seed sequences of miRNA families, we computed edit distances (Levenstein) between each pair of seed sequences of the differentially expressed families. The distance between the seeds of co-regulated miRNA families was compared to the distance between the seeds of any two miRNA families chosen at random. We found that seeds of co-regulated families were on average more closely related than random pairs of seed sequences (*P *< 0.001, see Methods and Additional file 7).

### Predicted targets of differentially expressed miRNAs

The presence of two reciprocal categories of differentially expressed mRNA and miRNAs suggests that miRNAs can regulate inversely expressed mRNAs. We also found that co-expressed miRNAs with the same differential expression profile often were encoded in the same genomic locations and had related seed regions (see above). Taking these observations into account, we theorise that targets of co-expressed miRNAs are likely to be also co-expressed and functionally related.

In order to test these propositions we have first obtained targets from TargetScan [21] for the families of 101 loosely defined down-regulated and 97 up-regulated miRNAs. We then tested if the expression of predicted targets is likely to be inverse to the expression trend of the respective miRNAs. Computational methods of miRNA target predictions are prone to false positive predictions [2]. To reduce the noise we only analyzed targets among the top 35% most highly expressed genes and excluded any targets predicted for both down and up-regulated miRNAs at the same time (see Methods). In this way 317 and 356 highly expressed and distinct targets were predicted for down- and up-regulated miRNAs respectively. As anticipated, predicted targets of down-regulated miRNAs were on average up-regulated. Conversely, targets of up-regulated miRNAs were on average down-regulated between the first and the last timepoints. The expression trends (slopes) of the members of these two categories of predicted targets were significantly different (Wilcox test *P *< 0.038, see Methods).

It should be noted that inverse expression of miRNAs and their targets is not the only possible mode of miRNA-target interaction. In a small number of biological systems correlation, rather than inverse expression, of miRNAs and their targets has been previously demonstrated for a number of miRNAs [42,43]. However, during the developmental timecourse, including nervous tissue development, correlation of the expression of miRNAs and their targets has been found to be either limited to a minority of miRNA-target interactions [44] or to be a feature of a late post-natal developmental onset [45]. In accordance with this, we observed that at least among highly expressed mRNA genes, reciprocal expression is a predominant characteristic of predicted miRNA-target interactions.

We tested whether specific biologically related gene sets could be found enriched in the two categories of predicted targets we obtained from TargetScan. We assessed enrichment of GO terms from Biological Process, Molecular Function and Cellular Compartment types of ontology [16]. Gene Ontology enrichment analysis showed that highly expressed predicted targets of down-regulated miRNAs were most enriched in proteins with intracellular/cytoplasmic localizations. The sole enriched term from the Biological Process category was 'protein transport'. Predicted targets of up-regulated miRNAs were on the other hand predominantly enriched in 'organelle' localized proteins, with three out of four enriched terms in the Biological Process category being 'RNA splicing', 'chromatin modification' and 'mRNA processing' (see Methods and Additional file 8 for the GO term enrichment analysis).

PSP coding genes are critically important for functionality of individual synapses and brain as a whole [17-19], therefore we specifically investigated if these genes are enriched among miRNA targets. A significantly high proportion of PSP genes are up-regulated during culture development (see above). We therefore asked whether up-regulated PSP genes were predicted to be targeted by differentially expressed miRNAs. We found that up-regulated PSP genes were significantly over-represented among the targets of down-regulated miRNA genes (*P *< 0.002), and not among the targets of up-regulated miRNAs. Interestingly, among the 27 up-regulated PSP genes (see Additional file 9) predicted to be targeted by down-regulated miRNAs, six were previously demonstrated to be involved in neuronal differentiation and synaptogenesis (Cdk5 [46], Gprin1 [47], Atp1a2 [48], Cend1 [49], Cacng2 [50] and Myo6 [51]).

In the top 35% most highly expressed genes (see Methods) we also performed a network analysis of the inversly expressed predicted targets using Reactome [52]. We identified 179 up-regulated genes targeted by down-regulated miRNAs and 189 down-regulated genes targeted by up-regulated miRNAs (see Methods). We observed enrichment for a number of pathways and subnetworks for many genes (see Additional file 10 for the full list of the predicted target genes in the significantly enriched subnetworks and Additional file 11 for the graphical representation of the results).

Targets of down-regulated miRNAs were enriched in several metabolic pathways. Numerous metabolic categories were found to be up-regulated during the course of the primary culture development (see above). This result serves as another indication that down-regulated miRNAs may regulate the transition to mature neuronal metabolism. Significant enrichment of targets in 'Signalling by NGF' pathway genes, supports this hypothesis, as NGF signalling is implicated in the survival of mature neurons [53]. Genes important for synaptic transmission are also predicted to be targeted by down-regulated miRNAs. For example, three members of 'Synaptic transmission' subnetwork were among the targets of down-regulated miRNAs (Fnbp1, Myo6, Cacng2), although this enrichment was not significant. At the same time the 'Membrane Trafficking' subnetwork was significantly enriched (*P *= 1.70*e *- 03).

Network analysis of the targets of up-regulated miRNAs generally recapitulates the results obtained with GO enrichment analysis (see above). Thus, subnetworks 'Processing of Capped Intron-Containing Pre-mRNA', 'Gene Expression', 'Transcription' were found to be significantly enriched (see Additional data file 10). In addition to that, we also identified enrichment for 'DNA Repair' and 'Telomere Maintenance' pathways. These findings further support the hypothesis that up-regulated miRNAs are involved in termination of DNA replication and finalizing switching to the established gene expression program, presumably the switch between the progenitor to mature neuronal cell program.

We predict the role of down-regulated miRNAs to be the targeting of many up-regulated mRNA coding genes, including many synaptic genes. Therefore in the adult brain these miRNAs are likely to be depeleted from the synaptic fraction, where many of the targets must be present at relatively high levels. We observed that the list of the four most down-regulated miRNAs in our study is identical to the four miRNAs most depleted in a synaptic fraction of the adult mouse forebrain [54]. This agrees with independent results obtained previously, using a different system and supports our hypothesis regarding the function of differentially regulated miRNAs.

## Discussion

We found that most miRNA genes, similar to protein coding genes, exhibited only two major modes of differential expression during differentiation and synaptogenesis in E17.5 mouse primary neuronal cultures. One set of miRNAs was continuously decreasing while another was continuously increasing in expression levels. Analysis of mRNA coding genes and miRNAs displaying these two trends showed that miRNAs were expressed inversely to their highly expressed predicted targets. This inverse relationship is consistent with previous results in other systems [12].

Those miRNAs showing common expression profiles were found to cluster within genomic loci. In total, 47% of co-expressed miRNAs were localized within just five genomic clusters on chromosomes 12, 14 and X. It was previously shown that miRNA genes localized in the same genomic cluster can be co-transcribed, co-regulated, share evolutionary origin and have common functions [39,40,55]. Thus it is likely that many co-expressed miRNAs in neuronal cultures have related functionality. The fact that a significantly high proportion of miRNAs, which are co-expressed in our study, have closely related seed regions supports this view.

Through a combination of mRNA and miRNA expression profiling and computational target prediction, we studied the regulatory potential of expressed miRNAs. It has previously been shown that miRNA regulation can be observed at both the mRNA [1,56] and protein level [2,11] and that there is reasonable agreement between the two.

Co-expression of genomically co-localized miRNAs and the apparent relatedness of their seed regions suggested using a combinatorial approach for functional analysis of predicted targets. The combined list of predicted targets of down-regulated miRNAs showed a significant enrichment in PSP genes. This implies that miRNAs developmentally down-regulated at earlier stages participate in the repression of a number of key neuronal genes at the time when neuronal network activity is undetectable [4,20]. This is consistent with finding the enrichment of cytoplasmic localization, protein binding and transport activity GO terms among the targets of the down-regulated miRNAs. On the other hand, targets of up-regulated miRNAs are not enriched in the PSP genes. Additionally, nuclear localization, RNA splicing and chromatin modification GO terms are enriched among the targets of up-regulated miRNAs (see Additional file 8 for GO terms list). Thus it is possible that this category of targets was involved in the reprogramming of progenitor gene expression to a differentiated neuronal cell expression, which naturally occurs during the tested developmental time-window. This view is further supported by pathway/network enrichment analysis and suggests that miRNA regulation is important for the organization of large sets of the neuronal proteome at different stages of development.

In addition to differentially expressed miRNAs we have identified a third distinct type of miRNA expression which is the steady-state expression at relatively high levels. Many of the miRNAs that were previously linked to neuronal biology (e.g. mmu-miR-124, mmu-miR-125 family, mmu-miR-137, mmu-miR-128, mmu-miR-9 and mmu-let-7) [6,25-32,57,58] belong to this category. Interestingly, we found that steady-state highly expressed miRNAs were predominantly located in isolated genomic loci rather than situated in genomic clusters of more than two miRNAs. We also found words complementary to the seed regions of at least three steady-state highly expressed miRNAs to rank among the most enriched words in 3'UTRs of relatively lowly expressed genes. Another 13 steady-state highly expressed miRNAs left a clear imprint on the global mRNA expression profile: A strong depletion signal for the seed-matching words of these miRNAs was observed in the 3'UTRs of highly expressed genes. These findings indicate that targets of many steady-state highly expressed miRNAs are likely to reside among relatively lowly expressed genes. Such categories of putative targets are likely to include many non-neuronal genes (e.g. immune system genes) and also genes involved in neuronal development at earlier timepoints (e.g. progenitor maintenance factors).

## Conclusion

Computational predictions of miRNA targeting are prone to produce false positive results and experimental validation is needed before a definitive conclusion is reached in any particular case. Nevertheless, this large scale study provides a global view on miRNA activity in neuronal cultures, which would be missing in a single gene approach. We identified three classes of miRNAs with distinct properties and characterized their possible targets. This may be important for the design of future knockout or other perturbation experiments and gives a starting point in the elucidation of the roles of hundreds of miRNAs in the development of neuronal cultures and hence synaptogenesis and formation of neuronal networks.

## Methods

### Primary Neuronal Cultures and RNA preparation

C57BL/6 c/c mice at 17.5 days of pregnancy were sacrificed and forebrains of embryos were dissected. Cell plating and culture manipulation were done as previously described [4]. At days one, two, four and eight of cultivation, total RNA was extracted using the miRNeasy Qiagen extraction kit in accordance with the manufacturer protocol. All mice were treated in accordance with the U.K. Animals Scientific Procedures Act of 1986, and all procedures were approved through the British Home Office Inspectorate.

### Enrichment of Gene Ontology (GO) terms

Enrichment of GO terms in mRNA expression clusters of differentially regulated genes and miRNA predicted targets was performed using the GOstats Bioconductor package [59]. All mouse Entrez genes were used as the test universe. The Hypergeometric test p. value cutoff was set to 0.001 and the 'conditional test' parameter was set to 'TRUE'.

### mRNA microarray experimental setup and initial data analysis

mRNA transcripts were profiled on Illumina Sentrix BeadChip Array Mouse-WG6_v1.1 microarray platform. For mRNA profiling we obtained total RNA samples from five biological replicates for day one, and six for each of the days two, four and eight. All replicates prior to normalization had high pairwise Pearson correlation values (r ≥ 0.99, not shown). Raw data were transformed and normalized using a variance-stabilizing transformation and robust spline normalisation [60,61]. Analysis of differential expression was done using the *limma *package available via Bioconductor [62,63]. When identification of differentially expressed genes was necessary, a p. value cutoff of 0.05 (not adjusted) and no magnitude of fold change cutoff was used in all cases to filter mRNA microarray probes. For cluster analysis of differentially expressed genes, median intensity values of all biological replicates per timepoint for each of the filtered probes was clustered using MCL with inflation parameter *I *= 3 [14,15]. Microarray probes were mapped to Ensembl gene and transcript identifiers using Ensembl Biomart via the R interface [64] to Ensembl Release 51 [65]. Where the derived ranking of identifiers was ambiguous due to multiple probes annotated to one gene or transcript, the probe with the lowest adjusted p. value of differential expression was selected. Where probes were annotated to multiple transcripts, the transcript with the longest 3'UTR sequence was selected.

### miRNA microarray experimental setup and initial data analysis

miRNA expression was profiled on Illumina Universal Sentrix Array Matrix. For miRNA microarry profiling we obtained total RNA samples from four biological replicates for day one, two for day two, four for day four and five for day eight. All replicates prior to normalization had high pairwise Pearson correlation values (r ≥ 0.95, not shown). Raw data were transformed using *log*_2 _transformation and normalized using quantile normalization as previously suggested for Illumina miRNA microarray experiments [66]. Analysis of differential expression was done using the *limma *package available via Bioconductor [63]. For identification of differential expressed miRNAs two types of filtering were applied: 1) Loose - using the significance of differential expression p. value cutoff 0.05 and no fold change cutoff; 2) Stringent - using both a p. value cutoff and fold change magnitude cutoff of 1.5. MCL clustering of differentially expressed miRNAs was performed with the inflation parameter *I *= 3 [14,15]. miRNA microarray probe identifiers were mapped to miRBase [41] (release 12) mature and hairpin miRNA identifiers using sequences supplied in the Il-lumina platform annotation file. Alignment of these sequences and miRBase sequences was done using SSAHA2 [67].

### Quantitative RT-PCR

Quantitative RT-PCR was performed with TaqMan miRNA primers and reagents according to the manufacturer manual. The Ct value was defined as the number of cycles at which fluorescence reached the level of five standard deviations above the baseline level of fluorescence between the cycles 3 to 15. Expression of snoRNA202 [68] was used as a control to obtain ΔCt values.

### Signalling Pathway Analysis

Progenitor signalling pathway gene lists were assembled from the literature [34-38], hand-curated and the gene-names were supplied to the TargetScan website (version 4.2).

### Analysis of word distribution in 3'UTRs

All mRNA microarray probes were sorted according to mean expression across the experimental time-course (from high to low). The sorted list of probes was translated into a sorted list of Ensembl transcript identifiers as described above. 3'UTRs were retrieved for transcript identifiers from Ensembl database. Where more than one 3'UTR sequence was assigned to a transcript, the longest sequence was selected. The Sylamer algorithm was then applied to calculate the distribution of enrichment of words complementary to seed regions at position 2 - 8 of profiled miRNAs in 3'UTRs of sorted transcripts [22]. Word depletion/enrichment was estimated by calculating hypergeometric p. value in bins growing by 250 sequences. Biases introduced by non-random distributions of sequences smaller than the size of a word (7 nucleotides) were controlled using Markov chain correction of order 3. For enriched and depleted words *log*_10 _of the p. value was plotted with a positive and negative sign respectively. If the p. value of depletion/enrichment for a given word was more significant than for at least 95% of other investigated words (e.g. lines in shades of red compare to gray lines in Figure 2) then depletion/enrichment was defined significant for this word.

We also used depletion/enrichment p. value for ranking of words. For this the same sorting of mRNA 3'UTRs was used and Sylamer was applied with the same parameters as above (except using 1000 identifiers per step of a growing bin). The top 10 and 20 most depleted/enriched were taken per all bins. As the next step, a Wilcoxon rank-sum test was performed on expression ranks of miRNAs with seed regions complementary to top depleted/enriched words against ranks of all profiled miRNAs. If the identified selection of miRNAs was expressed at a lower level than all profiled miRNAs on average, then *log*_10 _of Wilcoxon rank-sum test p. value was plotted with a negative sign, and with a positive sign if higher than average.

### Genomic clusters of miRNA genes

Only miRNA genes whose precursor sequences could be unambiguously mapped to the genome were used. Coordinates of miRNA precursor sequences mapped to mouse genome were obtained from miRBase release 12 [41]. A miRNA gene was defined as differentially expressed if the sequence of a strictly defined differentially expressed mature miRNA was aligned to it (see Methods). If more than one mature miRNA was aligned to a single precursor, then it was defined as either up- or down-regulated if at least one of the aligned mature miRNAs belonged to one of the two major expression categories (up- or down-regulated). It should be noted that we have not found an instance where several inversely expressed mature miRNAs were mapped to one precursor sequence (this was also true for loosely defined differentially expressed miRNAs). A genomic cluster of miRNA genes was defined as a group of genes whose neighbouring members were no further than 10 Kb apart and were transcribed from one strand and not separated by protein coding genes.

### Estimation of edit distance between miRNA seed regions

miRNA families conserved across human, mouse, rat, dog, and chicken were obtained from the TargetScan website [21] (version 4.1). A miRNA family was annotated as up- or down-regulated if it least one of the members was up- or down-regulated under a strict definition of differential expression. It should be noted that all families were dedicated to one mode of expression, i.e. we did not find an instance of a family which would contain strictly de-fined inversely expressed miRNA members at the same time. Seed regions (positions 2 - 8) of miRNAs in conserved families were also obtained from the TargetScan website. Edit distance was computed between any two pairs of miRNA seeds. Identical pairs (i.e. pairs of seeds with 0 distance) were excluded. The distribution of distances between all seeds was compared with the distribution of distances between the seeds of co-expressed miRNA families (distances between up- and down-regulated were computed separately and pooled together). The difference between the two distributions was assessed using a t-test.

### Analysis of predicted targets

From the TargetScan website [21] we obtained two lists of targets predicted for 101 down-regulated and 97 up-regulated miRNAs. Any targets present in both lists were excluded. To further reduce the noise associated with miRNA target prediction only targets among 35% of differentially regulated genes with highest average expression were considered (317 targets of down-regulated and 356 targets of up-regulated miRNAs). For each of the targets a ratio of expression values at day one divided by day eight was calculated. Median value of ratios for the targets of down-regulated miRNAs was on average 0.9878 (i.e. majority of targets are increasing in expression), while the ratio of for targets of up-regulated miRNAs was 1.0069 (i.e. a majority of targets is decreasing in expression). The Wilcoxon rank-sum test of the difference between the two sets of ratios was 0.03706. This result was confirmed by performing 10,000 random samplings of two groups of genes of the same size (317 and 356 genes) from 35% most highly expressed genes giving an empirical *P *≤ 0.0371.

For network and pathway analysis we used Reactome [52]. From the two groups of predicted target genes (317 targets of down-regulated and 356 targets of up-regulated miRNAs) we selected targets which were inversely expressed with respect to a target miRNA category. In this manner we identified 179 up-regulated genes targeted by down-regulated miRNAs and 189 down-regulated genes targeted by up-regulated miRNAs. The two lists of predicted targets were uploaded to the Reactome *Skypainter *website [69] and subnetwork enrichment was conducted using the default parameters. The lowest P. value of a child subnetwork was used to annotate the respective root pathway.

## Authors' contributions

AE, SG and SM conceived the study, participated in its design and coordination and drafted the manuscript. SM performed experiments and computational data analysis. All authors read and approved the final manuscript.

## Supplementary Material

Additional file 1 **GO terms enriched in mRNA expression clusters**. Top 15 most enriched GO terms in the two largest mRNA expression clusters (i.e. with average trends continuously decreasing or increasing between any two consecutive timepoints). Types of ontologies tested (*Ont*.): *BP *- biological process; *MF *- molecular function; *CC *- cellular component. Analysis results listed: *q *- number of genes from a GO term present in a group of predicted targets, *m *- total number of genes within a GO term, *P *- non-adjusted p. value for a given enrichment. See Methods for details. Click here for file

Additional file 2 **miRNAs belonging to the three major expression categories (with fold change expression cutoff)**. List of miRNAs by type of expression with a P. value cutoff of 0.05 and magnitude of fold change cutoff of 1.5. *Steady-state *- highly expressed miRNAs (among top 30 most highly expressed) showing no change of abundance between any timepoints. *Decreasing *- miRNAs classified by MCL (see Methods) into an expression cluster with average trend decreasing between all consecutive timepoints. *Increasing *-miRNAs classified by MCL into an expression cluster with average trend increasing between all consecutive timepoints. Numbers in parentheses show the ranking of a miRNA among all profiled miRNAs sorted by the maximal value of expression at any timepoint. Click here for file

Additional file 3 **miRNAs belonging to the three major expression categories (with no fold change expression cutoff)**. List of miRNAs by type of expression with a p. value cutoff of 0.05 and no magnitude of fold change cutoff. *Steady-state *- highly expressed miRNAs (among top 30 most highly expressed) showing no change of abundance between any timepoints. *Decreasing *- miRNAs classified by MCL (see Methods) into an expression cluster with average trend decreasing between all consecutive timepoints. *Increasing *-miRNAs classified by MCL into an expression cluster with average trend increasing between all consecutive timepoints. Numbers in parentheses show the ranking of a miRNA among all profiled miRNAs sorted by the maximal value of expression at any timepoint. Click here for file

Additional file 4 **RT-PCR validation of the three miRNAs profiled by microarrays**. (*A*) mmu-let-7a showed no change of abundance between the first (day 1) and the last (day 8) timepoints (-1 < ΔΔ*Ct *< 1). (*B*) mmu-miR-143 showed significant decrease (ΔΔ*Ct *< -4.43, P < 2*e *- 16) and (*C*) mmu-miR-370 a significant increase (ΔΔ*Ct *= 2.93, *P *< 2.1*e *- 19) in abundance levels. The error-bars are equal to two standard deviations of ΔCt values between the replicates. Click here for file

Additional file 5 **miRNAs among the top 100 most expressed with seed matching sites most enriched in the 3'UTRs of sorted genes**. *3*'*UTR words *- words enriched in 3'UTRs of sorted genes. *Complementary miRNA *- miRNA with seed region (positions 2..8) complementary to the enriched word. *miRNA expression trend *- average trend of miRNA expression category and cluster as defined by differential expression analysis and classified by MCL where applicable (see Methods): *steady-state *- highly expressed miRNAs (among the top 30 most highly expressed) showing no change of abundance between any timepoints; *decreasing *- miRNAs from an expression cluster with average trend decreasing between all consecutive timepoints; *increasing *- miRNAs from an expression cluster with average trend increasing between all consecutive timepoints; *other *- miRNAs classified into other minor MCL expression clusters. Numbers in parentheses show the ranking of a miRNA among all profiled miRNAs, sorted by the maximal value of expression at any timepoint. Click here for file

Additional file 6 **List of all miRNAs in five large genomic clusters enriched in differentially regulated miRNAs**. *Increasing Expression *- list of miRNAs from two genomic clusters on Chromosome 12. These clusters are enriched in miRNA with an average expression trend increasing between all consecutive time-points. *Decreasing Expression *- list of miRNAs from a genomic cluster on Chromosome 14 and two on Chromosome X enriched in miRNA with an average expression trend decreasing between all consecutive timepoints. Numbers in parentheses show chromosomal coordinates of the first and last miRNAs in a genomic cluster. Letters in parentheses refer to the expression of a miRNA: (*I*) - average trend increasing between all consecutive timepoints; (*D*) - average trend decreasing between all consecutive timepoints; (*s*) -no significant differential expression; (*e*) - average trend is not included in the above categories. Available information concerning associated genomic features and potential regulatory sites: *TSS *- transcription start site; *CpG *- CpG island evidence; *cDNA *- experimental EST evidence; *polyA *- polyadenylation evidence; *Regulatory Information *- experimental evidence from functional genomics database of Ensembl. Click here for file

Additional file 7 **The edit distance between the seed regions of co-expressed miRNAs**. Seed regions of co-expressed miRNAs were on average more similar than seed regions of randomly chosen miRNAs. All calculated edit distances (possible values from 1 to 7) are displayed as a histogram of percentages for pairs of co-expressed miRNAs (red bars) and randomly chosen pairs of miRNAs (grey bars). The distance distribution is shown with orange (co-expressed miRNAs) and grey (randomly chosen miRNAs) solid lines. Means of distance distributions are shown with orange (co-expressed miRNAs) and grey (randomly chosen pairs of miRNAs) dashed lines. Click here for file

Additional file 8 **GO terms most enriched (P *<*0.001) in highly expressed predicted targets**. Types of ontology terms tested (*Ont*.): *BP *- biological process; *MF *- molecular function; *CC *- cellular component. The results for targets exclusively predicted for the two types of miRNA expression clusters (with a decreasing or increasing average trends of expression) are given separately. Analysis results listed: *q *- number of genes from a GO term present in a group of predicted targets, *m *- total number of genes in a GO term, *P *- not adjusted p. value for a given enrichment. See Methods for details. Click here for file

Additional file 9 **miRNA targets among post-synaptic genes**. The list of genes coding for components of post-synaptic proteome (PSP) that were predicted to be targeted by miRNAs down-regulated during culture development (see Discussion for details). Click here for file

Additional file 10 **List of Reactome subnetworks for genes targeted by miRNAs**. Root Reactome subnetworks significantly enriched (unadjusted p. value *<* 0.05) among inversely correlated predicted targets (see Methods). *Subnetwork Category *- the name of a Root Reactome subnetwork with any of the children significantly enriched; *Enrichment P value *- unadjusted enrichment p. values; *Present/Total *- number of genes from the subnetwork category present in the query versus total number of genes in the subnetwork category; *Ensembl gene IDs *- Ensembl gene IDs of present genes; *Gene Symbols *- gene symbols of present genes. Click here for file

Additional file 11 **Graphical representation of Reactome subnetworks of genes targeted by miRNAs**. Individual Reactome events (reactions and/or pathways) with participating genes present among predicted targets of up- and down-regulated miRNAs (see Predicted targets of differentially expressed miRNAs). Every event is shown as an arrow, events with the genes present in the sets of miRNA targets are in black. Click here for file

## References

[B1] Lim LP, Lau NC, Garrett-Engele P, Grimson A, Schelter JM, Castle J, Bartel DP, Linsley PS, John-son JM (2005). Microarray analysis shows that some microRNAs downregulate large numbers of target mRNAs. Nature.

[B2] Baek D, Villén J, Shin C, Camargo FD, Gygi SP, Bartel DP (2008). The impact of microRNAs on protein output. Nature.

[B3] Giraldez AJ (2005). MicroRNAs Regulate Brain Morphogenesis in Zebrafish. Science.

[B4] Valor LM, Charlesworth P, Humphreys L, An-derson CNG, Grant SGN (2007). Network activity-independent coordinated gene expression program for synapse assembly. Proceedings of the National Academy of Sciences.

[B5] Mody M, Cao Y, Cui Z, Tay KY, Shyong A, Shimizu E, Pham K, Schultz P, Welsh D, Tsien JZ (2001). Genome-wide gene expression profiles of the developing mouse hippocampus. Proc Natl Acad Sci USA.

[B6] Sempere LF, Freemantle S, Pitha-Rowe I, Moss E, Dmitrovsky E, Ambros V (2004). Expression profiling of mammalian microRNAs uncovers a subset of brain-expressed microRNAs with possible roles in murine and human neuronal differentiation. Genome Biol.

[B7] Miska EA, Alvarez-Saavedra E, Townsend M, Yoshii A, Sestan N, Rakic P, Constantine-Paton M, Horvitz HR (2004). Microarray analysis of microRNA expression in the developing mammalian brain. Genome Biol.

[B8] Krichevsky AM, Krichevsky AM, King KS, Donahue CP, Khrapko K, Kosik KS (2003). A microRNA array reveals extensive regulation of microRNAs during brain development. RNA.

[B9] Dogini DB, Ribeiro PAO, Rocha C, Pereira TC, Lopes-Cendes I (2008). MicroRNA expression profile in murine central nervous system development. J Mol Neurosci.

[B10] Bayer SA, Altman J (1991). Neocortical development.

[B11] Selbach M, Schwanhausser B, Thierfelder N, Fang Z, Khanin R, Rajewsky N (2008). Widespread changes in protein synthesis induced by microRNAs. Nature.

[B12] Huang J, Babak T, Corson T, Chua G, Khan S (2007). Using expression profiling data to identify human microRNA targets. Nat Meth.

[B13] Parkinson H, Kapushesky M, Kolesnikov N, Rustici G, Shojatalab M, Abeygunawardena N, Berube H, Dylag M, Emam I, Farne A, Holloway E, Lukk M, Malone J, Mani R, Pilicheva E, Rayner TF, Rezwan F, Sharma A, Williams E, Bradley XZ, Adamusiak T, Brandizi M, Burdett T, Coulson R, Krestyani-nova M, Kurnosov P, Maguire E, Neogi SG, Rocca-Serra P, Sansone SA, Sklyar N, Zhao M, Sarkans U, Brazma A (2009). ArrayExpress update-from an archive of functional genomics experiments to the atlas of gene expression. Nucleic Acids Res.

[B14] van Dongen S (2000). Graph Clustering by Flow Simulation. PhD thesis.

[B15] Freeman TC, Goldovsky L, Brosch M, van Don-gen S, Mazière P, Grocock RJ, Freilich S, Thorn-ton J, Enright AJ (2007). Construction, visualisation, and clustering of transcription networks from microarray expression data. PLoS Comput Biol.

[B16] Ashburner M, Ball CA, Blake JA, Botstein D, Butler H, Cherry JM, Davis AP, Dolinski K, Dwight SS, Ep-pig JT, Harris MA, Hill DP, Issel-Tarver L, Kasarskis A, Lewis S, Matese JC, Richardson JE, Ringwald M, Rubin GM, Sherlock G (2000). Gene ontology: tool for the unification of biology. The Gene Ontology Consortium. Nat Genet.

[B17] Husi H, Ward MA, Choudhary JS, Blackstock WP, Grant SG (2000). Proteomic analysis of NMDA receptor-adhesion protein signaling complexes. Nat Neurosci.

[B18] Collins MO, Husi H, Yu L, Brandon JM, Anderson CNG, Blackstock WP, Choudhary JS, Grant SGN (2006). Molecular characterization and comparison of the components and multiprotein complexes in the postsynaptic proteome. Journal of Neurochemistry.

[B19] Genes to Cognition (G2C). http://www.genes2cognition.org/.

[B20] Siebler M, Koller H, Stichel C, Moller H (1993). Spontaneous Activity and Recurrent Inhibition in Cultured Hippocampal Networks. Synapse.

[B21] Lewis B, Burge C, Bartel D (2005). Conserved Seed Pairing, Often Flanked by Adenosines, Indicates that Thousands of Human Genes are MicroRNA Targets. Cell.

[B22] van Dongen S, Abreu-Goodger C, Enright A (2008). Detecting microRNA binding and siRNA off-target effects from expression data. Nat Meth.

[B23] Arora A, Simpson DA (2008). Individual mRNA expression profiles reveal the effects of specific microRNAs. Genome Biol.

[B24] Gennarino V, Sardiello M, Avellino R, Meola N, Maselli V, Anand S, Cutillo L, Ballabio A, Banfi S (2009). MicroRNA target prediction by expression analysis of host genes. Genome Res.

[B25] Cao X, Pfaff SL, Gage FH (2007). A functional study of miR-124 in the developing neural tube. Genes & Development.

[B26] Visvanathan J, Lee S, Lee B, Lee JW, Lee SK (2007). The microRNA miR-124 antagonizes the anti-neural REST/SCP1 pathway during embryonic CNS development. Genes & Development.

[B27] Makeyev EV, Zhang J, Carrasco MA, Maniatis T (2007). The MicroRNA miR-124 promotes neuronal differentiation by triggering brain-specific alternative pre-mRNA splicing. Molecular Cell.

[B28] Yu JY, Chung KH, Deo M, Thompson RC, Turner DL (2008). MicroRNA miR-124 regulates neurite outgrowth during neuronal differentiation. Exp Cell Res.

[B29] Silber J, Lim DA, Petritsch C, Persson AI, Mau-nakea AK, Yu M, Vandenberg SR, Ginzinger DG, James CD, Costello JF, Bergers G, Weiss WA, Alvarez-Buylla A, Hodgson JG (2008). miR-124 and miR-137 inhibit proliferation of glioblastoma multiforme cells and induce differentiation of brain tumor stem cells. BMC medicine.

[B30] Zhang Y, Chao T, Li R, Liu W, Chen Y, Yan X, Gong Y, Yin B, Qiang B, Zhao J, Yuan J, Peng X (2008). MicroRNA-128 inhibits glioma cells proliferation by targeting transcription factor E2F3a. J Mol Med.

[B31] Ferretti E, Smaele ED, Miele E, Laneve P, Po A, Pelloni M, Paganelli A, Marcotullio LD, Caffarelli E, Screpanti I, Bozzoni I, Gulino A (2008). Concerted microRNA control of Hedgehog signalling in cerebellar neuronal progenitor and tumour cells. EMBO J.

[B32] Laneve P, Marcotullio LD, Gioia U, Fiori ME, Fer-retti E, Gulino A, Bozzoni I, Caffarelli E (2007). The interplay between microRNAs and the neu-rotrophin receptor tropomyosin-related kinase C controls proliferation of human neu-roblastoma cells. Proc Natl Acad Sci USA.

[B33] Smirnova L, Grafe A, Seiler A, Schumacher S, Nitsch R, Wulczyn FG (2005). Regulation of miRNA expression during neural cell specification. Eur J Neurosci.

[B34] Mizutani K, Saito T (2005). Progenitors resume generating neurons after temporary inhibition of neurogenesis by Notch activation in the mammalian cerebral cortex. Development.

[B35] Raballo R, Rhee J, Lyn-Cook R, Leckman JF, Schwartz ML, Vaccarino FM (2000). Basic fibroblast growth factor (Fgf2) is necessary for cell proliferation and neurogenesis in the developing cerebral cortex. J Neurosci.

[B36] Qiu R, Wang X, Davy A, Wu C, Murai K, Zhang H, Flanagan JG, Soriano P, Lu Q (2008). Regulation of neural progenitor cell state by ephrin-B. J Cell Biol.

[B37] Guillemot F (2007). Cell fate specification in the mammalian telencephalon. Prog Neurobiol.

[B38] Stuckmann I, Weigmann A, Shevchenko A, Mann M, Huttner WB (2001). Ephrin B1 is expressed on neu-roepithelial cells in correlation with neocorti-cal neurogenesis. J Neurosci.

[B39] Baskerville S, Bartel DP (2005). Microarray profiling of microRNAs reveals frequent coexpression with neighboring miRNAs and host genes. RNA.

[B40] Xu J, Wong C (2008). A computational screen for mouse signaling pathways targeted by mi-croRNA clusters. RNA.

[B41] Griffiths-Jones S, Saini HK, van Dongen S, Enright AJ (2008). miRBase: tools for microRNA genomics. Nucleic Acids Res.

[B42] Shalgi R, Lieber D, Oren M, Pilpel Y (2007). Global and local architecture of the mammalian microRNA-transcription factor regulatory network. PLoS Comput Biol.

[B43] Martinez NJ, Ow MC, Barrasa MI, Hammell M, Sequerra R, Doucette-Stamm L, Roth FP, Ambros VR, Walhout AJM (2008). A C. elegans genome-scale microRNA network contains composite feedback motifs with high flux capacity. Genes & Development.

[B44] Tsang J, Zhu J, van Oudenaarden A (2007). MicroRNA-mediated feedback and feedforward loops are recurrent network motifs in mammals. Molecular Cell.

[B45] Liu H, Kohane IS (2009). Tissue and process specific microRNA-mRNA co-expression in mammalian development and malignancy. PLoS ONE.

[B46] Lagace DC, Benavides DR, Kansy JW, Mapelli M, Greengard P, Bibb JA, Eisch AJ (2008). Cdk5 is essential for adult hippocampal neurogenesis. Proc Natl Acad Sci USA.

[B47] Nakata H, Kozasa T (2005). Functional characterization of Galphao signaling through G protein-regulated inducer of neurite outgrowth 1. Mol Pharmacol.

[B48] Moseley AE, Lieske SP, Wetzel RK, James PF, He S, Shelly DA, Paul RJ, Boivin GP, Witte DP, Ramirez JM, Sweadner KJ, Lingrel JB (2003). The Na,K-ATPase alpha 2 isoform is expressed in neurons, and its absence disrupts neuronal activity in newborn mice. J Biol Chem.

[B49] Katsimpardi L, Gaitanou M, Malnou CE, Lledo PM, Charneau P, Matsas R, Thomaidou D (2008). BM88/Cend1 expression levels are critical for proliferation and differentiation of subven-tricular zone-derived neural precursor cells. Stem Cells.

[B50] Meng H, Walker N, Su Y, Qiao X (2006). Stargazin mutation impairs cerebellar synaptogenesis, synaptic maturation and synaptic protein distribution. Brain Res.

[B51] Osterweil E, Wells D, Mooseker M (2005). A role for myosin VI in postsynaptic structure and glutamate receptor endocytosis. J Cell Biol.

[B52] Matthews L, Gopinath G, Gillespie M, Caudy M, Croft D, de Bono B, Garapati P, Hemish J, Herm-jakob H, Jassal B, Kanapin A, Lewis S, Mahajan S, May B, Schmidt E, Vastrik I, Wu G, Birney E, Stein L, D'Eustachio P (2009). Reactome knowledgebase of human biological pathways and processes. Nucleic Acids Res.

[B53] Huang E, Reichardt L (2001). NEUROTROPHINS: Roles in Neuronal Development and Function. Annu Rev Neurosci.

[B54] Lugli G, Torvik VI, Larson J, Smalheiser NR (2008). Expression of microRNAs and their precursors in synaptic fractions of adult mouse forebrain. Journal of Neurochemistry.

[B55] Altuvia Y, Landgraf P, Lithwick G, Elefant N, Pfeffer S, Aravin A, Brownstein MJ, Tuschl T, Mar-galit H (2005). Clustering and conservation patterns of human microRNAs. Nucleic Acids Res.

[B56] Giraldez AJ, Mishima Y, Rihel J, Grocock RJ, van Dongen S, Inoue K, Enright AJ, Schier AF (2006). Ze-brafish MiR-430 promotes deadenylation and clearance of maternal mRNAs. Science.

[B57] Leucht C, Stigloher C, Wizenmann A, Klafke R, Folchert A, Bally-Cuif L (2008). MicroRNA-9 directs late organizer activity of the midbrain-hindbrain boundary. Nat Neurosci.

[B58] Shibata M, Kurokawa D, Nakao H, Ohmura T, Aizawa S (2008). MicroRNA-9 modulates Cajal-Retzius cell differentiation by suppressing Foxg1 expression in mouse medial pallium. J Neurosci.

[B59] Falcon S, Gentleman R (2007). Using GOstats to test gene lists for GO term association. Bioinformatics.

[B60] Lin SM, Du P, Huber W, Kibbe WA (2007). Model-based variance-stabilizing transformation for Illu-mina microarray data. Nucleic Acids Research.

[B61] Du P, Kibbe WA, Lin SM (2008). lumi: a pipeline for processing Illumina microarray. Bioinformatics.

[B62] Smyth G (2004). Linear models and empirical Bayes methods for assessing differential expression in microarray experiments. Stat Appl Genet Mol Biol.

[B63] Gentleman RC, Carey VJ, Bates DM, Bolstad B, Dettling M, Dudoit S, Ellis B, Gautier L, Ge Y, Gentry J, Hornik K, Hothorn T, Huber W, Iacus S, Irizarry R, Leisch F, Li C, Maechler M, Rossini AJ, Sawitzki G, Smith C, Smyth G, Tierney L, Yang JYH, Zhang J (2004). Bioconductor: open software development for computational biology and bioinformatics. Genome Biol.

[B64] Durinck S, Moreau Y, Kasprzyk A, Davis S, De B, Brazma A, Huber W (2005). BioMart and Biocon-ductor: a powerful link between biological databases and microarray data analysis. Bioinformatics.

[B65] Flicek P, Aken BL, Beal K, Ballester B, Caccamo M, Chen Y, Clarke L, Coates G, Cunningham F, Cutts T, Down T, Dyer SC, Eyre T, Fitzgerald S, Fernandez-Banet J, Graf S, Haider S, Hammond M, Holland R, Howe KL, Howe K, Johnson N, Jenk-inson A, Kahari A, Keefe D, Kokocinski F, Kule-sha E, Lawson D, Longden I, Megy K, Meidl P, Overduin B, Parker A, Pritchard B, Prlic A, Rice S, Rios D, Schuster M, Sealy I, Slater G, Smedley D, Spudich G, Trevanion S, Vilella AJ, Vogel J, White S, Wood M, Birney E, Cox T, Curwen V, Durbin R, Fernandez-Suarez XM, Herrero J, Hubbard TJP, Kasprzyk A, Proctor G, Smith J, Ureta-Vidal A, Searle S (2008). Ensembl 2008. Nucleic Acids Res.

[B66] Rao Y, Lee Y, Jarjoura D, Ruppert A, Liu C (2008). A Comparison of Normalization Techniques for MicroRNA Microarray Data. Stat Appl Genet Mol Biol.

[B67] Ning Z, Cox AJ, Mullikin JC (2001). SSAHA: a fast search method for large DNA databases. Genome Res.

[B68] Bak M, Silahtaroglu A, Moller M, Christensen M, Rath MF, Skryabin B, Tommerup N, Kauppinen S (2008). MicroRNA expression in the adult mouse central nervous system. RNA.

[B69] Reactome Skypainter website. http://www.reactome.org/cgi-bin/skypainter2.

[B70] Flavell SW, Cowan CW, Kim TK, Greer PL, Lin Y, Paradis S, Griffith EC, Hu LS, Chen C, Greenberg ME (2006). Activity-dependent regulation of MEF2 transcription factors suppresses excitatory synapse number. Science.

