# Supplementary figures and images for: Reciprocal regulation of microRNA and mRNA profiles in neuronal development and synapse formation
Source: BMC Genomics. 2009 Sep 8;10:419. doi: 10.1186/1471-2164-10-419 (PMC2759968; doi:10.1186/1471-2164-10-419)

mmu-let-7a

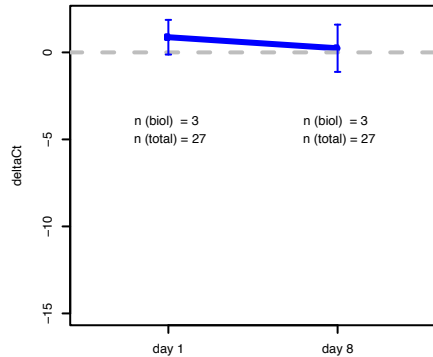

(A)

mmu-miR-143

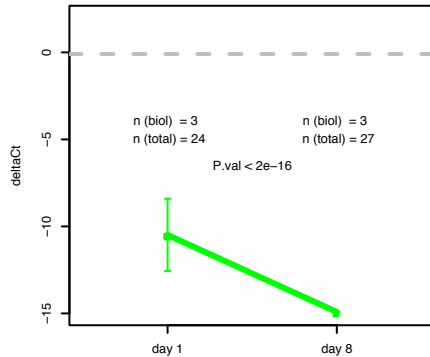

(B)

mmu-miR-370

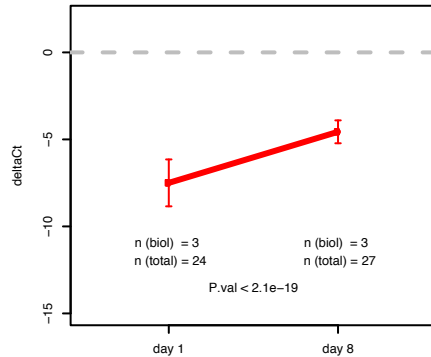

(C)

Supplement: Additional file 4 — RT-PCR validation of the three miRNAs profiled by microarrays. (A) mmu-let-7a showed no change of abundance between the first (day 1) and the last (day 8) timepoints (-1 < ΔΔCt < 1). (B) mmu-miR-143 showed significant decrease (ΔΔCt < -4.43, P < 2e - 16) and (C) mmu-miR-370 a significant increase (ΔΔCt = 2.93, P < 2.1e - 19) in abundance levels. The error-bars are equal to two standard deviations of ΔCt values between the replicates. [file 1471-2164-10-419-S4.pdf]

p-value < 0.001

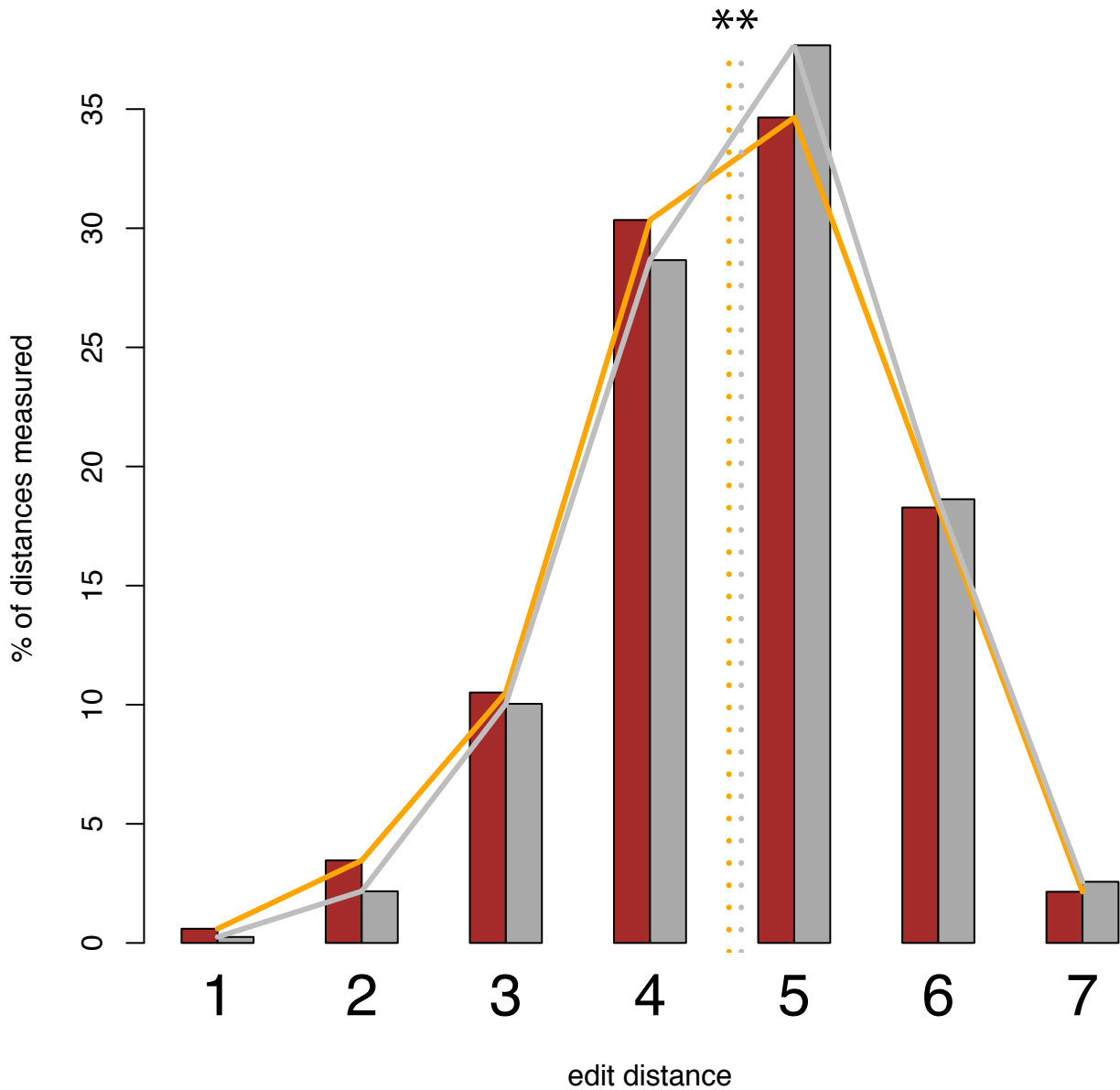

Supplement: Additional file 7 — The edit distance between the seed regions of co-expressed miRNAs. Seed regions of co-expressed miRNAs were on average more similar than seed regions of randomly chosen miRNAs. All calculated edit distances (possible values from 1 to 7) are displayed as a histogram of percentages for pairs of co-expressed miRNAs (red bars) and randomly chosen pairs of miRNAs (grey bars). The distance distribution is shown with orange (co-expressed miRNAs) and grey (randomly chosen miRNAs) solid lines. Means of distance distributions are shown with orange (co-expressed miRNAs) and grey (randomly chosen pairs of miRNAs) dashed lines. [file 1471-2164-10-419-S7.pdf]

## Targets of Down-Regulated miRNAs

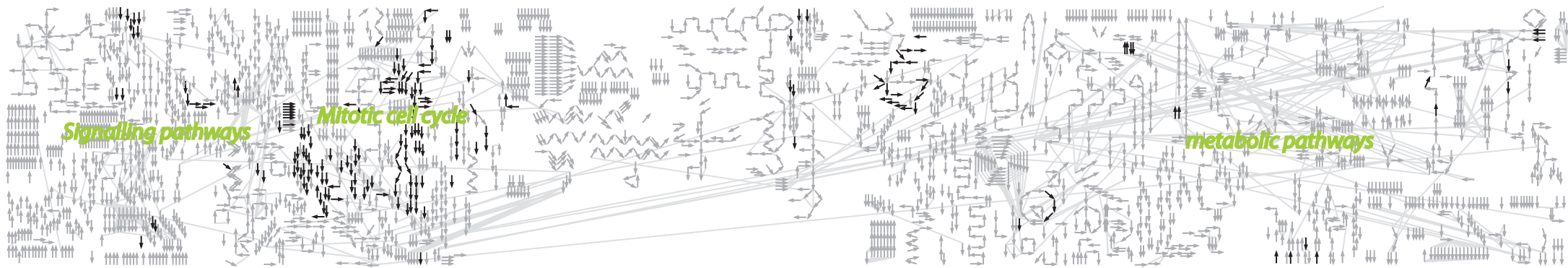

## Targets of Up-Regulated miRNAs

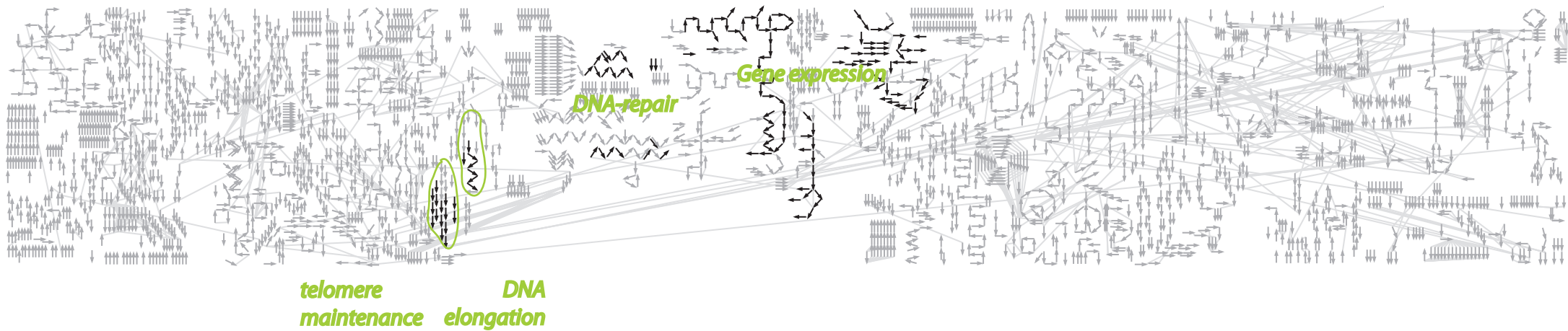

Supplement: Additional file 11 — Graphical representation of Reactome subnetworks of genes targeted by miRNAs. Individual Reactome events (reactions and/or pathways) with participating genes present among predicted targets of up- and down-regulated miRNAs (see Predicted targets of differentially expressed miRNAs). Every event is shown as an arrow, events with the genes present in the sets of miRNA targets are in black. [file 1471-2164-10-419-S11.pdf]
